# Curating global datasets of structural linguistic features for independence

**DOI:** 10.1038/s41597-024-04319-4

**Published:** 2025-01-18

**Authors:** Anna Graff, Natalia Chousou-Polydouri, David Inman, Hedvig Skirgård, Marc Lischka, Taras Zakharko, Chiara Barbieri, Balthasar Bickel

**Affiliations:** 1 Institute for the Interdisciplinary Study of Language Evolution (ISLE), University of Zurich, Zürich, Switzerland; 2 Department of Evolutionary Biology and Environmental Studies, University of Zurich, Zürich, Switzerland; 3 Institute for Mediterranean Studies, Foundation for Research and Technology - Hellas, Rethymno, Greece; 4 Department of Linguistic and Cultural Evolution, Max Planck Institute for Evolutionary Anthropology, Leipzig, Germany; 5 Institute of Mathematics, University of Zurich, Zürich, Switzerland; 6 Dipartimento di Scienze della vita e dell’ambiente, Università degli Studi di Cagliari, Cagliari, Italy

**Keywords:** Anthropology, Anthropology, Evolution of language

## Abstract

The increasing availability of cross-linguistic databases dedicated to documenting morphosyntactic, lexical and phonological features has proliferated the use of such data for studies on language evolution and human history. However, most of these databases were not designed to ensure independence of features, such that it is not valid to jointly use all their features in large-scale statistical analyses assuming independence of inputs. Here, we curate published data from five large linguistic databases to generate two global-scale cross-linguistic datasets: GBI (from the Grambank dataset), and TLI (using inputs from the World Atlas of Language Structures, AUTOTYP, PHOIBLE and Lexibank). The datasets minimize logical dependencies of features and forms of strong statistical dependencies that go beyond phylogenetic and geographical signal. They are also made available in densified form, reducing the proportion of missing data. We document our curation principles and workflows to ensure reusability of this framework with other inputs or thresholds of independence. Our curation steps on both datasets reveal robust and comparable global patterns of structural linguistic diversity.

## Background & Summary

Efforts to encourage Findable, Accessible, Interoperable, and Reusable (FAIR)^1^ datasets in comparative linguistics have promoted the adherence to cross-linguistic standards, such as using ISO 639-3 codes^2^ or glottocodes^3^ as unique language identifiers and Cross-Linguistic Data Formats (CLDF)^4^ for data structures. Making datasets available has also become easier thanks to Open Science publishing platforms such as Zenodo^5^. This facilitates the use of data from individual databases and data merging across databases - a welcome development for researchers addressing questions about structural linguistic distributions and their causes, particularly when independent extensive data collection for many features (also known as variables, parameters, characters, traits or properties) of linguistic structure is impractical.

Yet, when compiling published cross-linguistic data for statistical purposes, researchers should carefully evaluate the assumptions made by the methods used and curate the data accordingly. For instance, independence of inputs^6^ is presupposed by many statistical methods but not always given for linguistic data in terms of observations (here: languages, societies, etc.) or variables (here: linguistic features). Neglecting such considerations can render research outputs biased or even invalid, leading to potential misinterpretations^7,8^.

Linguistic researchers, especially typologists, have long been aware of the problem of non-independence of observations (languages), notably driven by relatedness and/or geographic proximity^9,10^, and have proposed possible solutions ranging from language sampling in a way that minimizes undesired biases^11^ to methods that model phylogenetic and/or spatial dependencies explicitly^12–15^. In contrast, non-independence of features has, with exceptions^16–18^, received less intensive and almost no systematic attention. Of course, specific universal or near-universal interfeatural associations among the world’s languages have long been of interest to linguists ^19,20^. However, the quest for such correlations among feature states has been de-emphasized in recent years: while some may be functionally grounded in aspects of human biology, cognition and/or sociality, others are the result of historical events such as contact or wide-scale spreads of language families and the attributes of their languages and do not represent generalized tendencies in human language *per se*^21^. As a consequence, dependencies (correlations, covariations) between features are currently less well established than spatial and phylogenetic dependencies, so they cannot, for instance, readily be used to inform covariance structures in multilevel (“mixed-effects”, “varying-effects”) models. Indeed, statistical methods so far generally fail to model dependencies between features. Therefore, a problem arises whenever analyses are carried out over multiple features simultaneously to allow for generalizations about entire languages rather than specific aspects of them. This includes applications such as the calculation of pairwise distances^17^, tests of linguistic area hypotheses^16^, or the bottom-up search for such areas^14^.

Since most of the available typological databases were not designed for specific statistical applications, the assumption of independence of features is usually violated even within a single database^22^. Thus, features included in a statistical analysis typically need to be evaluated for independence, variable by variable. Merging structural linguistic data from different databases requires even more careful consideration: on top of concerns about dependencies comes the fact that differing definitions in similar features limit the degree to which the datasets can be combined. The definition of even seemingly simple concepts such as grammatical gender or verbal tense can vary considerably. Therefore, extensive analysis of definitions and coding practices are crucial to assess whether features can validly be merged.

Here, we present methods and workflows with which researchers can identify and act on unwanted dependencies within and between typological databases, based on standard independence assumptions made by statistical methods. Specifically, we identified and removed logical and strong universal statistical dependencies between the morphosyntactic features of the Grambank database^15,23^, generating the GBI (“Grambank Independent”) datasets. We also assembled a second dataset, TLI (“Typology Linked and Independent”), using data from four distinct input databases: The World Atlas of Language Structures (WALS)^24^ with data on grammar, phonology and colexifications, AUTOTYP^25,26^ with chiefly morphosyntactic features, Lexibank^27,28^ with features on colexifications, and PHOIBLE^29^, which we used to generate phonological features.

**Fig. 1:**
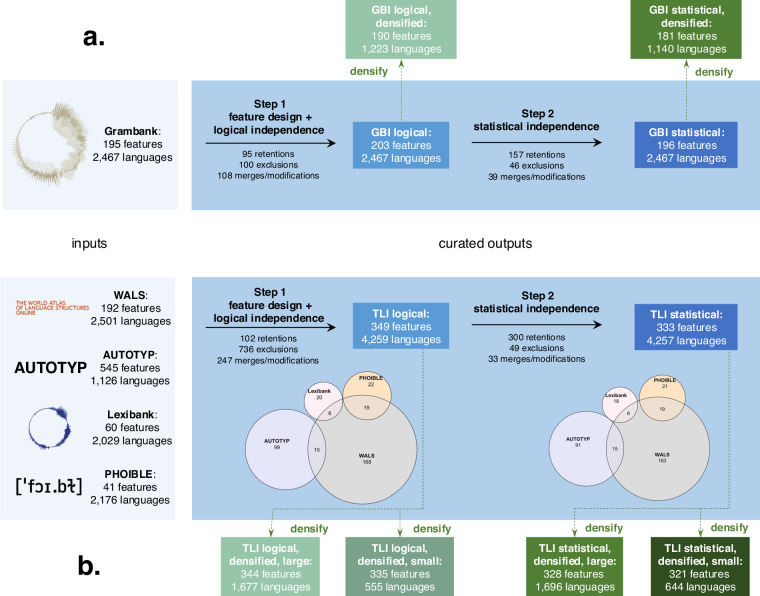
Overview of input data and curated outputs for (**a**) GBI (top) and (**b**) TLI (bottom). The graph includes the number of features and languages in each input dataset; the number of feature retentions, exclusions and merges/modifications for Step 1 (feature design + logical independence) and Step 2 (statistical independence at specific thresholds; see Methods); and the number of features and languages in the logical and statistical curations, as well as in the densified datasets.

Our curation procedure strives to maximize data retention of these input databases whilst minimizing dependence of features. For both GBI and TLI, we provide one curation of the data with minimized levels of logical dependence (see section Logical dependencies) and one curation where strong statistical dependencies (i.e., dependencies beyond shared inheritance and areality) are additionally removed (see section Statistical dependencies). While these steps resolve relevant problems for statistical analyses, they do so chiefly by introducing {NA} values in recoded features. This incurs problems for some applications. In response, we provide subsets of each curation that reduce the proportion of empty cells (see section Densification). Figure 1 provides an overview of all inputs and outputs presented here. While we hope that our curations directly contribute to a meaningful use of the data from the Grambank, WALS, AUTOTYP, Lexibank and PHOIBLE databases in multi-feature analyses, they also call for more detailed assessment of data assumptions when working with multi-variate datasets in language science.

## Methods

### Curation criteria and guiding principles

The GBI and TLI datasets were created to enhance the accessibility and interoperability of published data from five global typological databases for statistical frameworks that require independence of features and categorical features with internally coherent states. The GBI datasets result from curating Grambank, a resource that has the currently best balance between language coverage and scope in morphosyntactic features, with substantially fewer empty cells than any earlier linguistic database. The TLI datasets are the result of combining features from WALS, AUTOTYP, Lexibank and PHOIBLE into new datasets by collating original and creating independent modified (e.g., combined) features from all databases. TLI provides a set of global typological datasets with independent features, which, unlike the GBI datasets, are not restricted to the morphosyntactic domain, but also include information on phonology and colexification patterns. The features in GBI and TLI are not mere subsets of their respective input databases, but result from adopting a systematic data curation approach described below. We did not pursue a combination of Grambank or GBI and TLI because Grambank differs in feature definitions in many domains and has a different feature design (mostly binary features) in comparison to WALS and AUTOTYP (mostly multi-state features).

The GBI and TLI datasets were designed to align as well as possible with the data assumptions representative of many statistical methods, primarily relating to feature design (see section Feature design), logical independence (see section Logical dependencies) and statistical independence (see section Statistical dependencies). These assumptions are relevant in particular for methods that model many features together, e.g. through computing a distance metric across features, or through multivariate models of evolution and/or contact^14,30,31^.

On the level of feature design, most such methods assume that the features are categorical, and that the feature states can potentially be transmitted through inheritance or contact. In other words, states that are internally incoherent or varied, like “other” and “mixed”, violate the assumption (unless “mixed” is a specific, possible state). Such a principled definition of features and their states is also necessary to address hierarchical patterns, in which certain features are only applicable for certain observations, depending on other features^32^.

In addition to this, unless a feature dependency is explicitly modelled, it violates the independence assumption of any statistical method. In what follows, we focus on models that explicitly account for phylogenetic and spatial covariance structure but assume statistical independence between features. An obvious approach to remove dependencies for a multivariate analysis lies in extracting the independent outputs of an appropriate dimensionality-reduction technique (e.g., Principal Component Analysis (PCA) for continuous features, Factor Analysis of Mixed Data (FAMD) for a mix of categorical and numerical features, Multiple Correspondence Analysis (MCA) for categorical features). However, this removes all signal at the feature level, which in many cases is of central interest. Thus, if an analysis is to retain information at the feature level, interfeatural dependencies can and must be tolerated to some extent. The crucial question (to which there is no general answer) is to what extent and which kind of dependencies should be retained. Here, we answer this question as follows: we seek to eliminate all dependencies that are strictly logical (i.e., that by definition cannot have exceptions) because they provide no information gain and result in a series of problems for any statistical method (see below). Additionally, we eliminate those forms of probabilistic dependencies (i.e., statistical universals)^20,33^ that apply across language families and geographical macroareas and exhibit both few exceptions and a very high effect size.

To maximize the transparency and reusability of the data presented here, we identify and act on logical and statistical dependencies in two subsequent steps: we first treat logical dependencies (in tandem with feature design principles), yielding what we call “logically curated datasets”. Then, using these datasets as input, we identify and remove *a priori* expected and empirically confirmed (very) strong statistical dependencies among the logically curated features, yielding what we call “statistically curated datasets” (see Fig. 1). There are no intermediate datasets only curated for basic design and not logical dependencies or vice versa, since considerations regarding feature design and logical dependence usually have to be taken in tandem despite handling different aspects of the data.

Treating statistical dependencies in a separate step has two effects: first, researchers can use the logical curations of GBI and TLI for analyses that do not require the kind of statistical independence of inputs discussed here. Second, the thresholds of strong-enough statistical dependence can be adjusted if stricter (or laxer) criteria are preferred. In what follows, we provide concrete examples of non-optimal feature design, logical dependencies and statistical dependencies which are targeted in our workflows. We also show how our approaches generally identify and deal with these issues.

In addition to the assumptions regarding feature design and independence, we used three further criteria for data curation.

- Minimum feature coding: To reduce the complexity of the curation task, we only reviewed features coded (i.e., containing non-{NA} and non-{?} entries) for at least 100 languages, and we only included a curated feature if it exhibited at least 100 coded languages.
- Minimum variability: For a feature to be included in the curations, it had to exhibit a minimal degree of variability among coded languages: we considered features sufficiently variable if the second-most-frequent state is attested in at least three languages.
- Encoding of empty cells: Among cells lacking a state, we distinguish between those that are logically impossible to fill (coded “not applicable”, {NA}) and those where data is either missing because a language was not surveyed for a feature or explicitly coded as unknown in the source database (coded {?}). Although both {NA} and {?} states are treated equivalently by many software applications, this distinction is relevant for conceptual purposes, for imputation strategies, and for additional data collection.

For the many decisions of our workflow, we followed two guiding principles: Where several options were possible to optimize feature design or remove dependencies, we opted for those that allowed for maximal data retention (maximum data retention criterion), and that preserved as much of the original (input) dataset structure and design as possible (faithfulness criterion).

### Input data

#### Glottolog

The language identifiers used throughout this work are glottocodes from the most recent Glottolog release (v5.0)^34^. This data as well as metadata relating to geographical locations of languages was accessed via the file lang-metadata.csv, with coordinates and macroarea assignments obtained from Glottolog. Taxonomic data was also obtained from Glottolog via the R-package densify^35^. Missing coordinate and macroarea values were manually added to lang-metadata.csv.

#### Grambank

The Grambank (v1.0.3)^23^ database records data on 2,467 varieties for 195 grammatical typological features. All 2,467 languages are assigned to unique glottocodes. This full dataset served as input to the GBI datasets (see Fig. 1a).

#### WALS, AUTOTYP, Lexibank and PHOIBLE

The TLI datasets are the result of merging data from WALS on grammar, the lexicon (lexical classes and semantics) and phonology from three independent, global datasets: AUTOTYP, Lexibank and PHOIBLE. Thus, generating the TLI datasets required data from these four databases as inputs (see Fig. 1b).

From the latest WALS release (v2020.3)^24^, all 192 features were considered, yielding data on 2,501 languages. However, five glottocodes from this release (cypr1245, jogl1236, sanm1259, woro1255, and yidd1255) are no longer present in the latest Glottolog-release and had to be manually re-assigned to new glottocodes (mode1248, tase1235, sanm1295, worr1237, and east2295) based on their ISO 639-3 code^2^. One glottocode (kukn1238) has been assigned to the category of “bookkeeping” in Glottolog and was reassigned the updated glottocode dhod1238.

Amongst the datasets available in AUTOTYP (v1.1.2.)^25^ we selected the following 15:

- from the module Categories: Alienability, Clusivity, Gender and NumeralClassifiers;
- from the module Clause: ClauseWordOrder;
- from the module Morphology: MaximallyInflectedVerbSynthesis;
- from the module PerLanguageSummaries (which aggregrates what is coded at construction or morpheme levels): AlignmentForDefaultPredicatesPerLanguage, GrammaticalMarkersPerLanguage, LocusOfMarkingPerLanguage, MaximallyInflectedVerbAgreementAggregatedByMarkerPositionBinned4, MaximallyInflectedVerbInflectionAndAgreementCountsByPosition, MaximallyInflectedVerbInflectionCategoriesAggregatedPresence, MorphologyPerLanguage, NPStructurePerLanguage and PredicateClassesSemanticsPerLanguage.

The AUTOTYP datasets not considered either store metadata, encode information at the level of a construction or individual morphemes rather than a language or, in the case of the omitted aggregated datasets from the PerLanguageSummaries module, represent alternative aggregations to one of the included datasets for the same primary datasets. The included datasets contain 545 features and provide data on 1,126 languages, which are all mapped to the latest Glottolog release.

From Lexibank (v1.0)^28^, we considered all 60 structural features available in the lexicon and phonology datasets (i.e., features tracking full and partial colexifications), excluding only features providing metadata. This yields data on 2,029 languages attributed to unique glottocodes. One glottocode (lenc1244) is no longer present in the latest Glottolog-release and was manually re-assigned to the new glottocode lenc1239 based on its ISO 639-3 code. Four further glottocodes (naxi1246, sana1281, sout3125, and wela1234) were of the type “bookkeeping” and were manually assigned their updated glottocodes (yong1270, sana1298, sout2920 and ngal1291, respectively).

Finally, we used the segment inventory data from PHOIBLE (commit 7030ae0)^29^ to generate 41 binary phonological features (see Table 1) for all 2,176 languages available in the database that are attributable to unique glottocodes in the latest Glottolog release, selecting the largest inventory for a language if several were available. Five glottocodes (band1337, bubi1249, chua125, nucl1668, and samr1245) were of the type “bookkeeping” and were manually assigned the updated glottocodes (darl1243, bube1242, firs1234, kana1291, and cent2314, respectively). The features track the presence or absence of 41 classes of segments based on the vector of distinctive features descriptive of each segment present in the relevant segment inventory for each language. 17 of these features were defined to enrich data from WALS (e.g., the presence of uvular consonants), while 24 were defined independently of other databases to track further phonological features with global distributions that have been of interest to typologists (e.g., the presence of lateral consonants). The definitions of the features based on PHOIBLE’s notational conventions^29^ are available in the file phoible-feature-definitions.csv in the GitHub repository and the Zenodo archive^36^ hosting data and scripts for this project.

**Table 1:** Design ID and description of the binary segmental phonological features created from PHOIBLE for use in the TLI datasets.

Design ID	Feature description (presence/absence of)
D-PH4WA-01	uvulars
D-PH4WA-02	glottalized resonants
D-PH4WA-03	lateral obstruents
D-PH4WA-04	nasals
D-PH4WA-06	fricatives
D-PH4WA-07	velar nasals
D-PH4WA-08	nasalized vowels
D-PH4WA-09	front round vowels
D-PH4WA-10	interdentals
D-PH4WA-11	labial velars
D-PH4WA-12	pharyngeal epiglottals
D-PH4WA-13	dental alveolar voiced lateral approximant
D-PH4WA-14	implosives
D-PH4WA-15	ejectives
D-PH4WA-16	clicks
D-PH4WA-17	tone
D-PH4WA-18	bilabials
D-PHOIBLEBAF-01	affricates
D-PHOIBLEBAF-02	aspirated consonants
D-PHOIBLEBAF-03	aspirated fricatives
D-PHOIBLEBAF-04a	breathy vowels
D-PHOIBLEBAF-04b	breathy consonants
D-PHOIBLEBAF-05a	creaky vowels
D-PHOIBLEBAF-05b	creaky consonants
D-PHOIBLEBAF-06	glides
D-PHOIBLEBAF-07	liquids
D-PHOIBLEBAF-08	long consonants
D-PHOIBLEBAF-09	long vowels
D-PHOIBLEBAF-10	palatal laterals
D-PHOIBLEBAF-11	aspirated stops
D-PHOIBLEBAF-12	labialized consonants
D-PHOIBLEBAF-13	palatal and palatalized obstruents
D-PHOIBLEBAF-14	palatal and palatalized sonorants
D-PHOIBLEBAF-16	stridents
D-PHOIBLEBAF-17	laterals
D-PHOIBLEBAF-18	taps
D-PHOIBLEBAF-19	labiodentals
D-PHOIBLEBAF-20	coronal trills
D-PHOIBLEBAF-22	uvular trills
D-PHOIBLEBAF-23	alveolar approximants
D-PHOIBLEBAF-24	retroflex consonants

Linking all 838 raw input features for the generation of TLI via glottocodes yields data on 4,355 languages belonging to 355 families.

### Logically curated datasets

Here, datasets were curated both to eliminate strict logical dependencies and to ensure principled feature design.

#### Logical dependencies

Typological datasets can harbor many logical dependencies between features. Some of these dependencies are overt. For instance, WALS Chapter 30 A: Number of Genders^37^ has the states {None, Two, Three, Four, Five or more}, while WALS Chapter 31 A: Sex-based and Non-sex-based Gender Systems^38^ has the states {No gender system, Sex-based gender system, Non-sex-based gender system}. These two features share one state grouping together languages with no gender system. If a language has the state {None} for WALS 30 A, it logically follows that it will have state {No gender system} for WALS 31 A and vice versa. In an analysis assuming independence of features, this is problematic, because languages lacking gender will be deemed more similar to one another than languages that do have gender, since gender-free languages share the same state twice whilst languages with gender are not grouped for any feature. In this example, the dependence can be resolved by creating a new *meta-feature* (which can also be referred to as a macro or summary variable)^22^ tracking the presence of gender with the two states {Gender, No gender} from the input features. This new meta-feature can then be used to assign *not applicable* states (NA) in the conditioned subordinate features (also referred to as micro variables)^22^, which preserve the remaining typological information encoded in the original input features. In this instance, if the meta-feature state is {No gender}, this yields the state {NA} for the conditioned features derived from WALS 30 A and WALS 31 A, which track the number of genders (if gender exists) and whether or not the gender system is sex-based (if gender exists). Conditioning features on the state(s) of one or more meta-features is not only a way to resolve logical dependencies, but also results in more principled typologies, in which only relevant languages are surveyed for each feature.

#### Feature design

In addition to overt cases of logical feature state dependence (as in the case of gender in WALS above), similarities among languages can be boosted in a less obvious way. For instance, in the Grambank database there are thirteen binary {1, 0} features that track whether specific manifestations of gender/noun class systems are attested in languages worldwide (see Table 2). However, these features are not conditioned on the presence of a gender/noun class system. This has the effect that languages lacking a gender/noun class system will appear unduly similar in a cross-featural analysis, because they share 13 feature states under this setup. This boosting of similarity can be prevented by conditioning the input features on the presence of a gender/noun class, such that only languages with a gender/noun class system are surveyed for their specifics, with all other languages receiving the state {NA} in the conditioned features. Since the Grambank questionnaire provides no feature tracking the general presence of a gender/noun class system, we once again turn to the creation of a meta-feature. In this meta-feature, languages with state {1} for at least one of the features typologizing gender systems in the original Grambank questionnaire are assigned state {1}, languages with state {0} for all these features are assigned state {0} and all other languages (i.e., languages that are coded {?} for one or more features without being coded {1} for any features) are assigned state {?}. We opted for transfer of unknown states {?} to the meta-features to ensure that the meaning of {0} remains consistent as known absence.

**Table 2:** Grambank features relating to gender/noun class systems.

ID	Feature description
GB051	Is there a gender/noun class system where sex is a factor in class assignment?
GB052	Is there a gender/noun class system where shape is a factor in class assignment?
GB053	Is there a gender/noun class system where animacy is a factor in class assignment?
GB054	Is there a gender/noun class system where plant status is a factor in class assignment?
GB170	Can an adnominal property word agree with the noun in gender/noun class?
GB171	Can an adnominal demonstrative agree with the noun in gender/noun class?
GB172	Can an article agree with the noun in gender/noun class?
GB177	Can the verb carry a marker of animacy of argument, unrelated to any gender/noun class of the argument visible in the NP domain?
GB192	Is there a gender system where a noun’s phonological properties are a factor in class assignment?
GB198	Can an adnominal numeral agree with the noun in gender/noun class?
GB314	Can augmentative meaning be expressed productively by a shift of gender/noun class?
GB315	Can diminutive meaning be expressed productively by a shift of gender/noun class?
GB321	Is there a large class of nouns whose gender/noun class is not phonologically or semantically predictable?

Feature ID and description of 13 binary Grambank features representing a typology of gender/noun class systems across languages.

Another principle adopted here was to recode features that document two or more typological aspects simultaneously (henceforth: *compositional features*). An example of such a compositional feature is WALS Chapter 9 A: The Velar Nasal^39^. This feature tracks the global distribution of the velar nasal and has three states: {No velar nasal, Initial velar nasal, No initial velar nasal}. The feature not only documents the presence of the phoneme, but also its associated position (word-initial or not). This state space is not optimal for comparative analysis because the states exhibit a hierarchical relationship to one another: the position of the velar nasal logically presupposes its presence. In uncurated form, the three states of this feature would be treated as equally different from each other although they are not. In addition to this, the original encoding does not reflect the conceptualization present in the relevant WALS chapter^39^: “This distribution [of the velar nasal] has two unrelated aspects. One is the striking areal distribution of the presence vs. absence of phonemic ŋ among the languages of the world. The other striking aspect of phonemic ŋ is its phonotactic distribution.” This conceptualization can be directly encoded by splitting the feature into two. The first encodes whether a velar nasal is present at all, which otherwise is not explicitly encoded anywhere, merging the second and third state from the initial feature into a new, coherent state: {No velar nasal, Velar nasal}. The second feature then encodes whether languages have or lack an initial velar nasal. Logically, this feature is only applicable to languages with a velar nasal, resulting in the following states: {Initial velar nasal, No initial velar nasal, NA}. This decomposition makes the hierarchical relationship inherent in the original data explicit and maintains the original conceptualization of the feature. A compositional design can also be found in sets of binary features in Grambank. There are, for example, two separate features tracking different manifestations of plural number marking (GB044: *Is there productive morphological plural marking on nouns?* and GB318: *Is plural number regularly marked in the noun phrase by a dedicated phonologically free element?*). There are analogous features for singular, dual, trial and paucal number categories. These features all track two typological aspects in tandem: The presence of a number category and its degree of fusion with the noun. However, the presence of any number category itself (in either form) or whether number categories are ever marked fusionally (*on nouns*) or not (*by a dedicated phonologically free element*) cannot be tracked in this setup. We resolve this issue with meta-features that track the presence of each of the number categories and conditioned features that track whether languages ever display fusion to the noun vs. phonologically free marking of number. This way, we can track the overarching phenomena of interest that are combined across several compositional input features. Replacing (sets of) compositional input features by meta-features offers an alternative categorization of typological space that recomposes the feature space into its primary dimensions. This process, much like defining features in database design, is opinionated and dependent on the research project at hand. Here, this process was guided by co-designers of two of the input datasets, Hedvig Skirgård for Grambank and Balthasar Bickel for AUTOTYP, taking into account the typological framework of the databases.

A third feature design principle we adopted was to ensure that all included feature states should be coherent or cohesive. Hence, feature states of the type {mixed}, {intermediate}, {other} or similar were altogether avoided when possible or replaced by {?}.

#### Curation

**Fig. 2:**
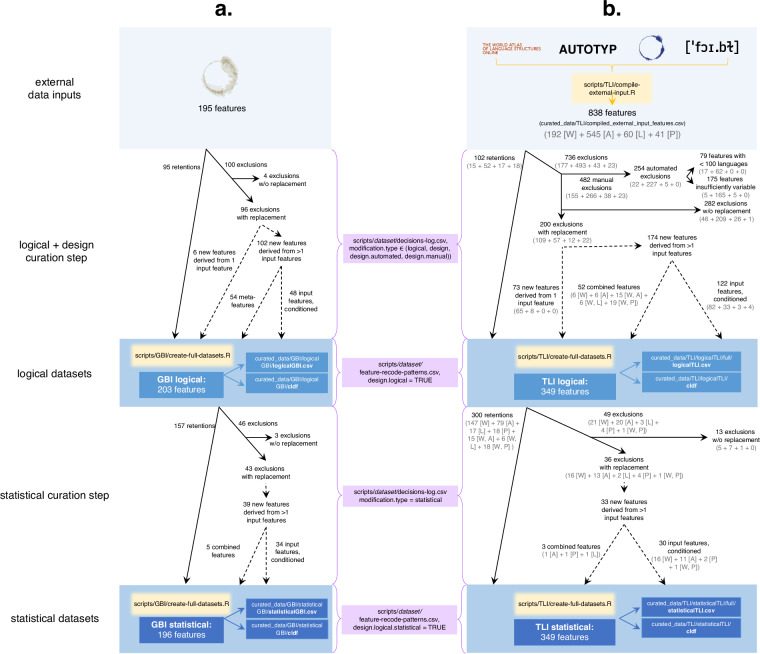
Flowchart for GBI (**a**) and TLI (**b**) depicting external data inputs (light blue), curation files (purple) and R scripts (yellow) required to generate the logically and statistically curated datasets (dark blue). The path to each file within the repository is provided. Blue arrows indicate access to curated data outputs. Black arrows indicate the treatment of input features in each curation step. Dashed arrows indicate the derivation of new features from input features. Entries in grey in (**b**) indicate the number of features represented from each respective origin database (WALS, AUTOTYP, Lexibank, PHOIBLE), for each documented step.

Figure 2 provides an overview of the curation process for both the GBI-logical and TLI-logical datasets. If an input feature did not align with the curation criteria outlined above, it was excluded from the dataset. This affected 100 features for GBI and 736 features for TLI. Most such features had to be manually identified. It was only possible to use automatic detection for a subset of the curation criteria, namely features coded for too few languages (here: 99 languages or fewer) or those exhibiting insufficient variability (here: if the second most frequent state of a feature is attested in only one or two languages). The application of the other curation criteria required direct human involvement, as it necessitated expert knowledge.

In many cases, it was possible to include a modified form of the excluded original input feature by deriving new features (such as the meta-features tracking the presence of velar nasals or gender/noun classes generally, as well as their conditioned features). This led to the creation of 108 new features for GBI and 247 new features for TLI. Such new features were the result of one or more of the following operations:

- exclusion and/or merging of some input feature states (6 in GBI, 73 in TLI), e.g., excluding a mixed or intermediate feature state, such as states denoting “no dominant word order” in features about noun phrase orders.
- merging an input feature with one or more other features either (1) to create a meta-feature, or (2) to create a feature combining data from two mergeable features (54 in GBI, 52 in TLI). Operations of type (1) were performed to introduce features tracking e.g., the presence of gender, case, and articles, while operations of type (2) allowed for the introduction of new features combining data on, e.g., the position of the nominal plural marker from WALS and AUTOTYP, the colexification of finger and hand in WALS and Lexibank, and the presence of uvulars in WALS and PHOIBLE. (When this type of feature was derived, the decision about which input feature would be prioritized in case of conflict was based on a case by case evaluation, taking into account feature definitions and number of data points per input feature. These decisions are documented in the appropriate decisions-log.csv file.)
- conditioning input features on the states of one or more other (meta-)features they logically depend on (48 in GBI, 122 in TLI), e.g., conditioning of a feature tracking whether articles can agree with the noun in gender on the presence of both articles and nominal gender.

**Fig. 3:**
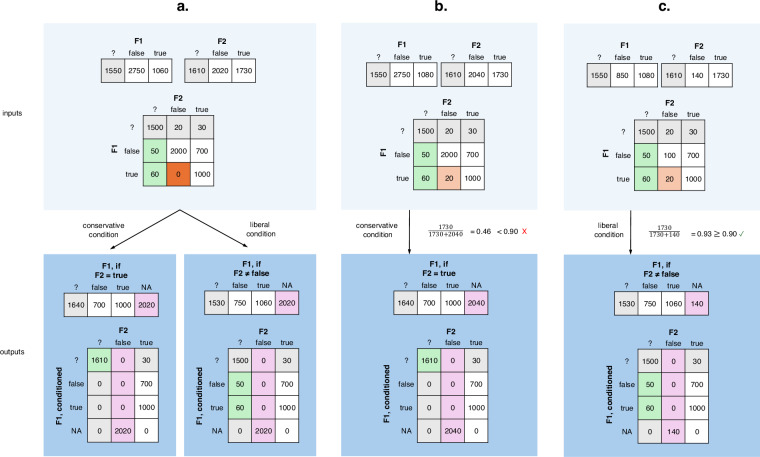
Differences between conservative and liberal conditioning, exemplified with hypothetical features F1 and F2 and implementations of conservative and liberal conditioning of F1 on F2 in different scenarios. The tabulations indicate the count of languages per feature state. (**a**) Illustration of conservative vs. liberal conditioning of a dependence between F1 and F2. In this example, F1 logically depends on F2: the state {true} for F1 presupposes state {true} in F2 (i.e., counts above 0 in dark orange cell are logically impossible). Any languages encoded {false} for F2 must therefore be assigned the state {NA} after conditioning F1 (pink cells). However, the conditioned feature can either be forced to be agnostic (conservative condition, left) about languages coded for F1 but not F2 (green cells), or the information available in the original feature F1 can be preserved (liberal condition, right) at the cost of inappropriately assigning some languages the state {false} rather than {NA}. (**b**) and (**c**) Illustration of conservative vs. liberal conditioning of a statistical dependence. Assume the statistical dependencies between F1 and F2, such that the state {true} in F1 strongly presupposes the state {true} in F2 (i.e., high counts in light orange cell are unlikely), meet our defined criteria for feature modification in the second curation step in (**b**) and (**c**). If the state of F2 that F1 is being conditioned on accounts for under 90% of languages, we perform conservative conditioning (**b**). If the state of F2 that F1 is being conditioned on is much more frequent than the other states and accounts for 90% or more of languages, we perform liberal conditioning in favor of the common state (**c**).

Conditioning refers to explicitly disconsidering in the coding of a feature the subset of languages for which this feature is irrelevant. It can be done in two ways, which we here call *conservative* and *liberal* conditioning (see Fig. 3). Suppose a feature F1 is only applicable to languages with some state (e.g., {true}) of feature F2. Under conservative conditioning we only consider languages that are explicitly coded as {true} for F2, while under liberal conditioning we also consider languages for which no information is available in feature F2. Liberal conditioning therefore includes more languages in a conditioned version of F1, at the expense of explicitly coding some languages that could as well be {NA}, which is not strictly accurate. If several features are liberally conditioned on the same feature, then dependencies may emerge between these features. We therefore generally performed conservative conditioning in the logical curation process. Because most features in logical relationships were coded for identical or very similar sets of languages, this did not result in considerable loss of information. We only conditioned liberally in exceptional cases when careful assessment of all relevant features made clear that this would not introduce unwanted dependencies among features.

The motivation underlying each modification is documented in detail in both a human- and machine-readable manner in decisions-log.csv, with a unique identifier. The identifiers of the modifications were assigned to the types “design” or “logical” for GBI (where this distinction was more straightforward) and “design.automated” or “design.manual” in TLI.

Separate tables for each dataset (feature-recode-patterns.csv) list, describe and define all features serving as inputs or derived in the curation process, linking each feature to all relevant modifications and specifying all recoding operations necessary to generate them. They also include mappings of each feature to five feature groupings (lexical semantics, lexical classes, grammatical categories, grammar and phonology, see Table 3). Custom-made functions were written in R^40^ to parse these tables and produce the language-feature matrix and logically curated datasets in CLDF. A flowchart of this process is provided in Fig. 2, and the R scripts are available on the GitHub repository and on Zenodo^36^. The structure underlying the feature-recode-patterns.csv files and parts of the R code parsing it were based on an earlier recoding of the WALS data^41^.

**Table 3:** Feature group definitions for GBI and TLI features.

Feature group	Description
Lexical semantics (Lexicon)	Relating to how meaning is structured and expressed in open-class, semantically rich, lexical items; e.g. questions of colexification or partial similarities between lexical items.
Lexical classes (Lexicon)	Relating to syntactically relevant information in open-class, semantically rich, lexical items; e.g. valency classes, alienability classes, gender systems, inflection classes, parts of speech.
Grammatical categories (Grammar)	Relating to presence and nature of grammatical categories without information on the specific expression of the category in the sentence or any lexical class it may build on; e.g. presence of passive, alienability distinctions, gender agreement, case, nominal vs verbal conjunctions.
Grammar (Grammar)	Relating to formal aspects of grammar; e.g. specific expression of gender on nouns, locus of marking, suppletion patterns, morphological behavior, linear ordering (flagged as: Grammar_linear_order, everything else is Grammar_other).
Phonology (Phonology)	Relating to phonological segments (consonants flagged as: Phonology_segmental_c; vowels flagged as: Phonology_segmental_v), prosody (Phonology_prosodic) or other aspects of phonology.

#### GBI-logical

In the creation of the GBI-logical dataset (see Fig. 2a), 95 of the 195 input features from Grambank were retained without any modification. 100 original Grambank features were excluded. Four of the excluded features (GB079, GB080, GB160 and GB400) were removed without replacement because they were tracking phenomena that were too internally diverse. Six features relating to word order were replaced by modified features, in which the state denoting “no single dominant word order” was excluded. The remaining 90 excluded features were replaced by meta-features derived from the input features and/or by features resulting from conditioning input features on meta-features. This led to the net creation of 102 derived features, 54 of which represent meta-features. 19 meta-features were created to address logical dependencies. The remaining 35 were created to resolve compositional features. Meta-data on the features and languages of the dataset are available in CLDF in the files parameters.csv and languages.csv (see Data Records).

Naming the GBI-logical features followed certain rules. We maintained identifiers of retained original Grambank features (e.g., GB046). Modified original features created by excluding an intermediate state were given the suffix *e* (e.g., GB025e). Meta-features were assigned a unique unused three-digit identifier and the suffix *m* (e.g., GB551m), while merged features created for resolving compositional features into their dimensionality recomposing features were assigned the suffix *drm* (e.g., GB851drm). Finally, we assigned conditioned features the suffix *c* (e.g., GB082c). This suffix was also assigned to merged features that were conditioned (e.g., GB553drmc).

#### TLI-logical

To produce the TLI-logical dataset (see Fig. 2b), 102 of the 838 input features from WALS, AUTOTYP, Lexibank and PHOIBLE were retained without modification, and 736 were excluded. 254 of these were automatically excluded as not complying with our curation criteria: 79 features were coded for fewer than 100 languages and 175 features did not exhibit sufficient variability. All other 482 excluded features were removed after being manually reviewed, 282 of them without replacement for reasons of redundancy. The remaining 200 excluded features were replaced by 247 new features. 73 of them are modified features derived from a single feature, 52 are combined features and 122 are input features conditioned on one or more other features they logically depend on. Meta-data on the features and languages of the dataset are again available in CLDF in the files parameters.csv and languages.csv (see Data Records).

For naming the TLI logical features we applied the following rules (Table 4): retained original input features were assigned the prefix *o*, followed by the database and input feature name. For WALS features, we used only the chapter number to refer to the feature. For AUTOTYP features, we also specified the module of origin. For features generated from PHOIBLE, we specified that the binary aggregated features (BAF) were derived from input inventories and not explicitly coded typological features. Examples for original feature names are thus: *o_WALS_1A, o_autotyp_Alienability_NPHasInalienableClasses, o_lexibank_CommonSubstringInBoyAndGirl* and *o_PHOIBLE_BAF_affricates_presence*. All recoded features were assigned the prefix *rec*, followed by an appropriate combination of prefixes to describe the operations performed to generate the feature. Prefix *e* thereby denoted the exclusion of one or more (intermediate or otherwise inappropriate) states (e.g., *rec_e_WALS_93A*), prefix *a* refers to other operations adapting (e.g., fusing) input feature states (e.g., *rec_a_WALS_108A*), prefix *c* refers to resolving a logical dependence by conditioning (e.g., *rec_c_WALS_29A*), prefix *m* followed by the relevant feature names separated by + denotes the merging of several input features (e.g., *rec_m+WALS_10A+PHOIBLE_BAF_nasalizedVowels_presence*), and prefix *d* accompanied by a suffixed number indicates the decomposition of an input feature (e.g., *rec_da_WALS_110 A_1* and *rec_da_WALS_110A_2*). Following this explicit naming scheme produced rather long, albeit semantically transparent, names for some features. Therefore, we also assigned each feature a shorter unique identifier that is less transparent, consisting of the prefix *TLI* followed by a unique combination of four digits.

**Table 4:** Naming conventions for features of the TLI dataset.

Feature name element	Interpretation
o_ prefix	original input feature
rec_ prefix	recoded feature
e_ prefix	exclusion of one or more feature states
a_ prefix	adaptation (e.g. fusion) of input feature states
m_ prefix	merging of several input features (separated by +)
+ separator	separates input features merged to generate new feature
d_ prefix … _number suffix	features from a decomposed compositional input feature
**Examples**	
rec_m + WALS_10A + PHOIBLE_BAF_nasalizedVowels_presence	new feature resulting from the combination of WALS feature 10A and PHOIBLE feature BAF_nasalizedVowels_presence
rec_da_WALS_110A_1, rec_da_WALS_110A_2	two features resulting from the decomposition of WALS feature 110A, that have also undergone adaptations of their states

### Statistically curated datasets

#### Statistical dependencies

If features that strongly depend on each other throughout the sample are included in an analysis which assumes feature independence, this may have problematic consequences for the results. Cross-featural dependencies may dominate the results in ways that obscure other meaningful relations. It is therefore important to identify cases of strong statistical non-independence and to act on them prior to certain types of statistical analysis. In what follows we offer a way of addressing this issue.

**Fig. 4:**
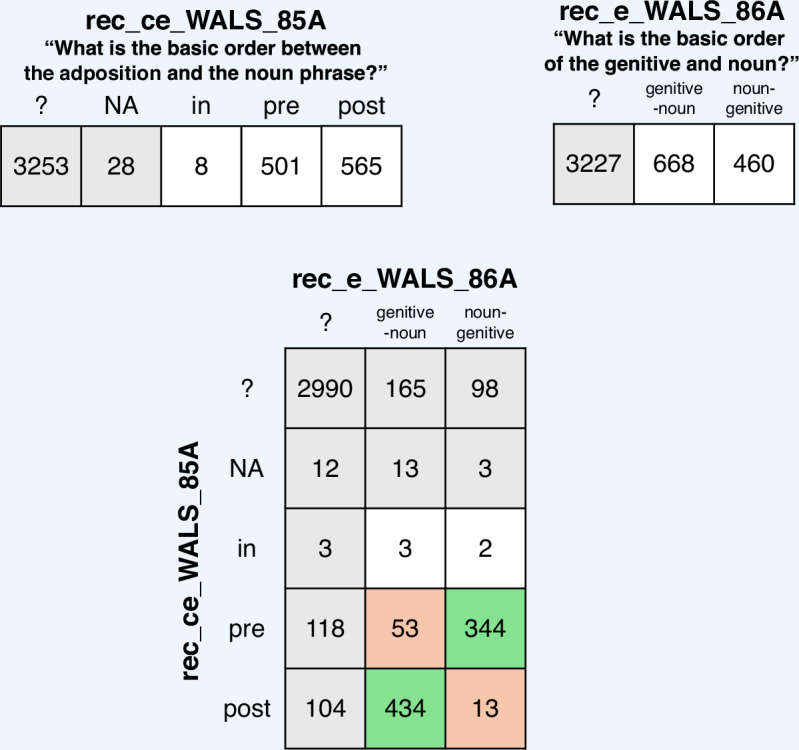
Number of languages per feature state and cross-table for features denoting the order of adposition and noun phrase vs. the order of genitive and noun. If Greenberg’s Universal 2 holds (“In languages with prepositions, the genitive almost always follows the governing noun, while in languages with postpositions it almost always precedes”^19^, cells in green should exhibit high counts, while cells in orange should exhibit low counts.

For instance, from a logical perspective, it is true that the order of different elements in the noun phrase are independent of one another. For example, it is possible for languages with prepositions and for languages with postpositions to have “genitives” (nouns functioning as modifiers in the same way as genitive-marked nouns) either preceding or following the noun. However, empirically, their distribution is strongly correlated: most languages with prepositions place their genitives after the noun, and most languages with postpositions place their genitives before the noun^19^. Figure 4 visualizes this general tendency in our data, showing the cross-table of the two adapted WALS features in the TLI-logical dataset tracking the order of these elements. Among those languages coded for both features, only 53 languages have the combination of prepositions and genitive-noun order and 13 have the combination postpositions and noun-genitive, while 344 languages have the combination prepositions and order noun-genitive and 434 languages have the combination postpositions and order genitive-noun. Overall, 778 languages behave as predicted by the putative universal, while there are only 66 exceptions to it. It is clear that in this example, the features examined are statistically non-independent. Many such dependencies have been noted in the literature^42^. They pose a problem for statistical analysis if they are not modelled. If they are modelled, for example phylogenetically^12,43^ and/or geographically^10,44^, they are not problematic. However, researchers may decide to tolerate even dependencies beyond phylogenetic and areal signal if they harbor a substantial number of exceptions that can be useful for linguistic or historical inferences.

#### Curation

**Fig. 5:**
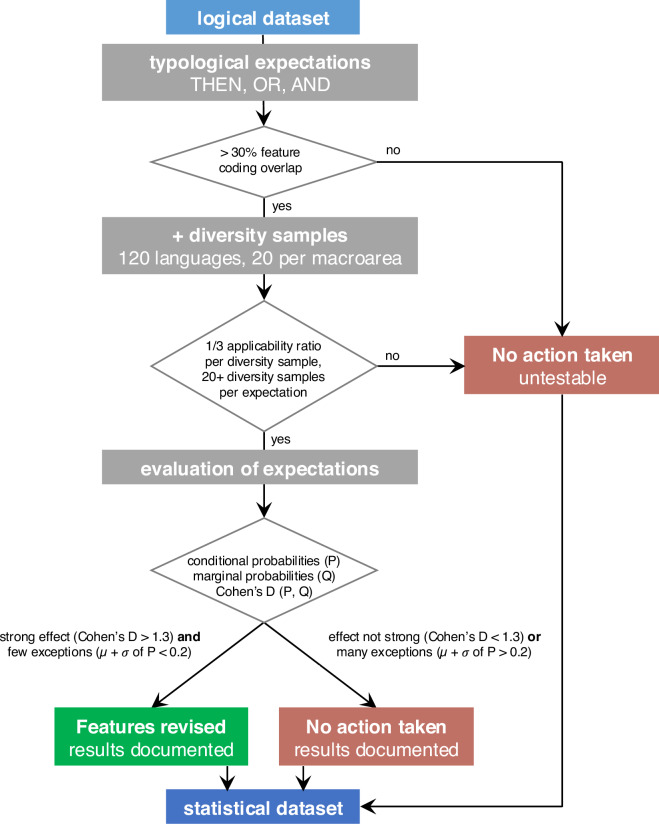
Overview of the statistical curation workflow described in the section Statistically curated datasets, curation.

The statistically curated datasets are the result of subjecting the logically curated datasets to an additional series of modifications to remove certain forms of statistical cross-featural dependencies: those that (1) are expected *a priori* and have a plausible underlying mechanism given current textbook knowledge in linguistic typology, in the sense that they can be readily understood as driven by some shared property; (2) hold universally across phylogenetic units and geographic macroareas; and (3) show a very strong effect with few exceptions. For an overview of the statistical curation workflow, see Fig. 5.

**Fig. 6:**
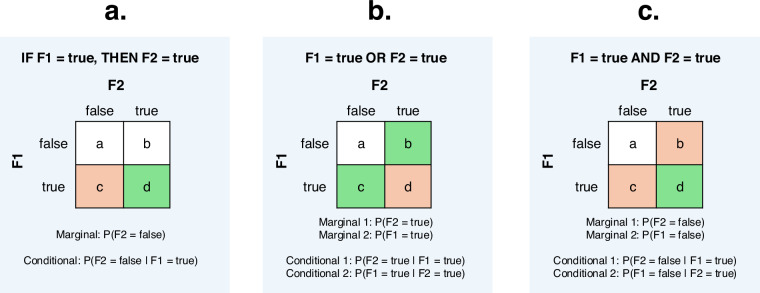
Illustrations of expectation types, including cells expected to contain high (green) and low (orange) counts. (**a**) THEN-type (unidirectional implication): In THEN-tests, the assumption is that *c* << *d* across diversity samples. The marginal probability P(F2 = false) should therefore be higher than the conditional probability P(F2 = false|F1 = true). (**b**) OR-type (exclusionary bi-directional implication): In OR-tests, the assumption is that *c >> d* and *b >> d* across diversity samples. Each marginal probability (P(F2 = true), P(F1 = true)) should therefore be higher than its corresponding conditional probability (P(F2 = true|F1 = true), P(F1 = true|F2 = true)). (**c**) AND-type (positive bi-directional implication): In AND-tests, the assumption is that *c << d* and *b << d* across diversity samples. Each marginal probability (P(F2 = false), P(F1 = false)) should therefore be higher than its corresponding conditional probability (P(F2 = false|F1 = true), P(F1 = false|F2 = true)).

To reduce the number of false positives, we did not conduct an automatic search of all possible correlations, but assessed the evidence for specific relationships between feature states based on expert knowledge^45^. We formulated all candidate correlation patterns (henceforth: expectations) as implicational universals and assigned them a modification identifier of the type “statistical”. The motivation behind each expectation was also recorded in the decisions-log.csv file of the dataset in question in human- and machine-readable form. The patterns to be investigated were either unidirectional (flagged as type THEN), exclusionary bidirectional (type OR) or positive bidirectional (type AND), and states assumed to be correlated positively or negatively were explicitly listed (Fig. 6).

Examples of THEN-expectations include, for GBI: *IF possessive classifiers present, THEN alienable/inalienable distinction present* (modification.ID: S-POS-7), and for TLI: *IF labiodentals present, THEN fricatives present* (modification.ID: S-PHO-18). For OR-expectations, examples include, for GBI: *bound comparative degree marker present OR non-bound comparative degree marker present* (modification.ID: S-CPA-2), and for TLI: *arm and hand colexified OR finger and hand colexified* (modification.ID: S-LEX-12). Finally, examples for AND-expectations include, for GBI: *transitive clause verb-initial AND intransitive word order VS* (modification.ID: S-WOO-06a), and for TLI: *suffixal case AND suffixal plural* (modification.ID: S-CASPLU-01).

Testing these expectations on raw contingency tables would be improper since languages in our sample are not independent observations. Patterns of shared inheritance and areality could cause spurious correlations or obscure genuine ones^9,10^. While it is debated how to best model these dependencies, we opted here for a solution that requires the least amount of information. Therefore, we generated multiple random independent language samples of 120 languages each, with 20 distinct lineages (families or isolates) from each of the six Glottolog macroareas (Africa, South America, North America, Eurasia, Papunesia and Australia)^11,46^. For GBI, which is densely coded, we generated 500 such diversity samples, sampling from all languages attested in the GBI-logical dataset. For TLI, we sampled only from languages coded for at least 10 features in the TLI-logical dataset and generated 1000 diversity samples. We did this to account for the sparsity in TLI.

**Fig. 7:**
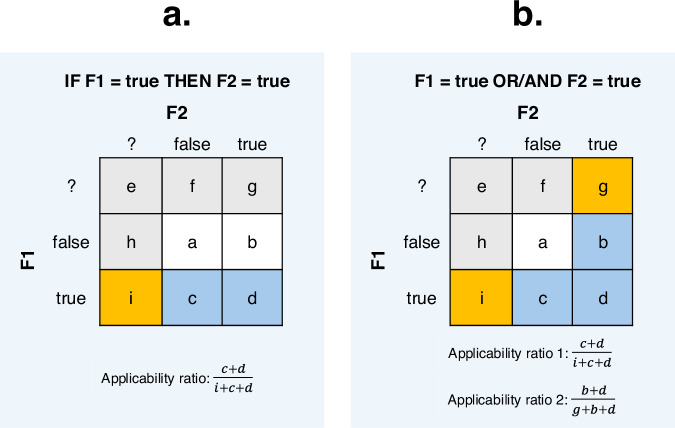
Definition of applicability ratios. (**a**) THEN-type (unidirectional implication). (**b**) OR- and AND-types (bi-directional implications). Diversity samples in which this ratio (THEN) or one of these ratios (OR/AND) was lower than 1/3 were not considered in the assessment of statistical dependencies.

For each expectation, we computed a set of measurements (see the decisions-log.csv files) to assess whether adjustments needed to be made to the data. Specifically, for each expectation, we first computed the number of languages that are for each of the features and the overlap between these. In cases with little language overlap (concretely, when the better-coded features have less than 30% of its languages coded for the other feature), we did not act on dependencies even if they were strong because they affect only a small set of languages, minimizing the violations of independence assumptions. For the expectations that cleared this bar, we computed several measures obtained from the cross-tabulation of the relevant feature states for each of the 120-language diversity samples. First, we calculated *applicability ratios* for each of the 120-language diversity samples (see Fig. 7). These represent the overlap of languages at the level of *feature states*, as opposed to the level of the *features* as a whole, which was considered for the previous step. For the expectations of the THEN type, we considered data points with the relevant state for the first feature, e.g. {true}. The applicability ratio is the proportion of these languages that are coded (e.g., {true, false}) for the second feature out of all the languages that are coded for the relevant state for the first feature (see Fig. 7a). If the number of {?} or {NA} datapoints is very high for the second feature, then the applicability ratio will be low, such that the dependency between states cannot be meaningfully assessed in the diversity sample in question. For the THEN-expectations, only one applicability ratio was calculated. For the bidirectional expectations (OR and AND, see Fig. 7b), two applicability ratios had to be computed (one for each direction). If the applicability ratio (or the lower of the two ratios) was below 1/3 for a given diversity sample, we removed this diversity sample for further computations to ensure our dependence assessments were based on samples informative for the relevant expectation. If fewer than 20 diversity samples remained available in this way, we considered that the expectation could not be tested reliably, and no further action was taken. All thresholds employed here (applicability ratio of 1/3, minimum of 20 diversity samples) are necessarily somewhat arbitrary. We defined them so that we only act on dependencies that we are confident to test and that affect substantial numbers of languages.

**Fig. 8:**
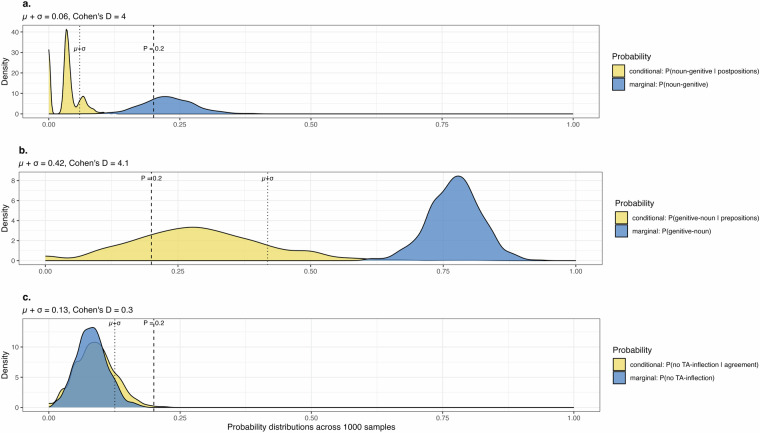
Examples of expectations in the TLI dataset and their results. (**a**) ‘if postpositions, then word order genitive-noun’, positive example (few exceptions, very strong effect size). (**b**) ‘if prepositions, then word order noun-genitive’, negative example 1 (many exceptions, very strong effect size). (**c**) ‘if verb agreement, then tense-aspect inflection’, negative example 2 (few exceptions, very weak effect size). Yellow: distribution across 1000 random diversity samples of the conditional probability (i.e. for a language to deviate from the formulated expectation); blue: distribution across 1000 random diversity samples of the baseline or marginal probability. (**a**) and (**b**) visualize probability distributions relating to Greenberg’s Universal 2, the bottom graph for Universal 30^19^. Given our thresholds, it is only in the first case that the probability distributions warrant modification for the statistical curation.

For all diversity samples that remained, we then computed the conditional probability (in the THEN-type, see Fig. 6a) or conditional probabilities (in the OR- and AND-types, see Fig. 6b,c) for a language to exhibit the unexpected combination(s) of feature states. To assess whether observed low probabilities were indeed the result of a dependency in feature states and not merely a consequence of skewed distributions in both features^47^ we also computed the corresponding marginal probability (for the THEN-type) or marginal probabilities (for the OR- and AND-types) under the tested expectation. We then computed the mean and standard deviation of all computed probability distributions across all relevant diversity samples and logged them in decisions-log.csv. Additionally, we computed and recorded the paired Cohen’s D statistic (Effect Size Index)^48^ of the vectors of the conditional and marginal probabilities using the function cohensD() in the lsr package^49^. We then determined which dependencies to act on according to two thresholds that were chosen to identify dependencies with strong effect size and few exceptions. We only acted on dependencies if Cohen’s D > 1.3, denoting very high effect size^50^, and if, additionally, µ + σ < 0.2 for the conditional probability (or probabilities). If µ + σ = 0.2, then no more than 20% of languages are exceptions to the expectation in ~84% of diversity samples. Figure 8 shows one example of an expectation meeting both these criteria and two examples of only one of the criteria being met. As above, these thresholds are – unavoidably – slightly arbitrary. Therefore, we recorded additional dependencies even when we did not act upon them as *known.remaining.dependencies* in feature-recode-patterns.csv. Specifically, we logged remaining dependencies where the effect size was very high (Cohen’s D > 1.3, but µ + σ ≥ 0.2. We also documented remaining dependencies where Cohen’s D > 1.3 for only one dimension of a bidirectional OR- or AND-test. Each threshold can of course be tailored to the requirements of any specific project.

To act on dependencies, we implemented similar methods as in the logical curation step: We identified which features had to be excluded and which of these could be replaced in a modified form to ensure maximum data retention. Most commonly, we performed feature conditioning to resolve dependencies. If the feature state which was to be conditioned upon was substantially more common than the other states (covering at least 90% of languages), we opted for liberal conditioning in favor of this common feature state to avoid data loss for the conditioned feature; otherwise, we performed conservative conditioning (see Fig. 3b,c). In a few cases, we also merged the interdependent features into a single new feature with states defined to combine information from both input features. We documented each modification in the relevant decisions-log.csv file alongside the entries of the associated statistical test. In some cases, features that were excluded participated in further expectations. If these features were replaced by modified features, we assessed the relevant expectations again using the modified features.

All resulting exclusions and additions are documented in the file feature-recode-patterns.csv, together with a general grouping of features (Table 3). Features are linked to their corresponding expectations documented in decisions-log.csv via modification IDs. We provide R scripts to generate the statistically curated datasets as language-feature matrices and as CLDF datasets (Fig. 2) on GitHub and Zenodo^36^. The script test-dependencies.R generates the diversity samples used and allows reproducing the test results in the respective decisions-log.csv file.

#### GBI-statistical

To create the GBI-statistical dataset (Fig. 2a), 157 of the 203 input features from GBI-logical were retained without modification. Of the 46 removed features, three were excluded without replacement and 43 were replaced by 39 new features, of which 34 were conditioned features and 5 were combined features. 84 dependencies did not clear our thresholds, so we did not act on them but logged them in the column known.remaining.dependencies of the parameters.csv file in the CLDF-version of the statistically curated datasets instead. Results are based on 210 expectations that we evaluated according to the procedure outlined above. Meta-data on the features and languages of the dataset are readily available in the CLDF-files parameters.csv and languages.csv (see Data Records).

The new feature identifiers were named as follows: identifiers of features already present in the GBI-logical dataset kept their name. Modified features were given the capitalized suffix *C* if they were conditioned (e.g., GB058C, GB953drmcC). Merged features received either the capitalized suffix *F* if their state space represented the four-state combination of the 2 × 2 input states (e.g., GB995F), *EO* if the new feature resulted from an OR-test and new feature states were the two mutually exclusive input states (e.g., GB900EO) or *EON* if the new feature resulted from an OR-test and new feature states were the two mutually exclusive input states and a neither state (e.g., GB332EON).

#### TLI-statistical

To create the TLI-statistical dataset (Fig. 2b), 300 of the 349 input features from the TLI-logical dataset were retained without modification. Of the 49 removed features, 13 were excluded without replacement, and 36 were replaced by 33 new features. 30 of these were conditioned input features and 3 were combined features. Additionally, 48 known remaining dependencies were found and can be retrieved in the column known.remaining.dependencies in the parameters.csv file in the CLDF-version of the statistically curated datasets. Results are based on the assessment of 250 expectations. Meta-data on the features and languages of the dataset are available in the CLDF-files parameters.csv and languages.csv (see Data Records).

Unchanged features retained their names from the TLI-logical dataset. New features received a capitalized prefix *C* (within their existing prefix if applicable) if they were conditioned (e.g., *rec_C_PHOIBLE_BAF_labiodentals_presence, rec_dccaC_WALS_37A_4*) and prefix *EON* if they were merged features resulting from an OR-test with the two mutually exclusive input states and a neither state (e.g., *rec_EON+lexibank_TreeInBark+lexibank_BarkAndSkin*). Again, alternative identifiers of the form *TLIXXXX* are available as shorter but non-transparent feature identifiers.

### Densification

The GBI and TLI datasets may be too large and/or too sparsely coded for a given application. For instance, computationally intensive Bayesian algorithms such as sBayes^14^ may take impractically long to converge with input datasets with over 2,000 languages (the GBI-statistical dataset), let alone with inputs containing over 4,000 languages (the TLI-statistical dataset). In such cases, users may consider reducing the dataset by excluding languages which are closely related to others in the sample. Some algorithms also run more efficiently if the proportion of missing data is minimized, and imputation methods usually require a certain minimal proportion of filled cells to perform successfully. Reducing the proportion of uncoded cells also enhances the usability of the datasets for distance-based applications.

**Fig. 9:**
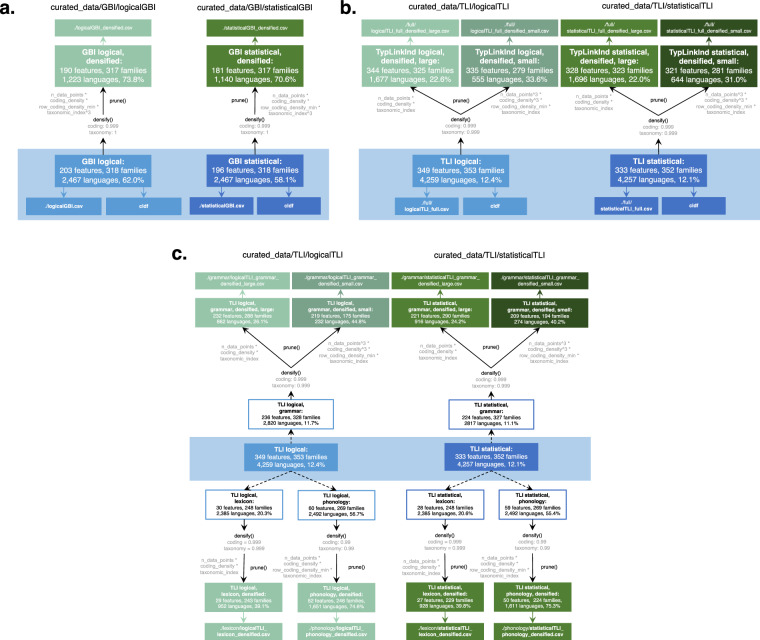
Densification flowchart for the GBI datasets (**a**), the full TLI datasets (**b**) and domain-wise subsets of the TLI datasets (**c**). Curated input data and their relevant subsets are depicted in blue. Densified datasets are depicted in green. Paths to each file in the repository are also provided. Densification steps are indicated by black arrows, with densification parameters depicted in grey.

To address these issues, we performed matrix densification on the logical and statistical GBI and TLI datasets using the R package densify^35^, making available densified versions of the datasets (Fig. 9a,b). For the TLI datasets, we additionally performed densifications on domain-specific subsets of the data, generating densified datasets with only grammatical, phonological, and lexical features respectively (Fig. 9c).

The functions of the densify package iteratively remove languages and features with low coding densities of any input matrix provided, thereby incrementally increasing overall coding density, and then identify the optimal *sub-matrix*. Both the pruning process and the identification of the optimal sub-matrix are modulated by user-defined parameters, which specify the relative weight given to the trade-offs between data retention, coding density, and taxonomic diversity of languages. In particular, the taxonomic diversity parameter allows users to reduce biases in the densified language sample driven by the lack of systematic documentation in certain families or branches.

Here, we opted for the following settings for all densifications: Pruning (via densify()) was performed using the default type of density mean in a taxonomy-sensitive setup, balancing taxonomic diversity and coverage. The minimum variability was set to 3, such that features counting fewer than three languages in their second-most-frequent state were dropped, mirroring the principle adopted in data curation. To identify the optimal sub-matrix (via prune()), we used different scoring functions depending on the specific dataset, as explained below. We consistently included the basic trade-off between the overall matrix coding density and the number of included data points (by specifying the same non-zero exponent to each of these measures in the scoring function) and consistently avoided penalizing sparsely coded features, because many missing states at the feature level were deliberately introduced to remove dependencies. The remaining scoring function characteristics varied across densifications and are specified and motivated below.

#### Densifying the GBI datasets

For the logical and statistical GBI datasets, we performed one densification procedure each (see Fig. 9a). Both input datasets are large (2,467 languages each) and fairly densely coded (62.0% and 58.1% filled cells, respectively). In general, both languages and features are densely coded. The aim of the densification was therefore mainly to exclude languages in order to reduce the size of the dataset. Because the input language sample does not represent the world’s language families evenly, we primarily focused on removing languages from well-represented language families. For iterative matrix densification, we specified the taxonomy weight to be slightly higher than the coding weight, specifically: density_mean_weights = list(taxonomy = 1, coding = 0.999). This ensured that densify() increased the weight given to taxonomic diversity in the iterative densification process. For matrix selection using prune(), we specified the taxonomic diversity of the language sample to weigh heavily: scoring_function = n_data_points * coding_density * row_coding_density_min * taxonomic_index^3. This yielded matrices retaining 99.7% of families. The densified logical matrix had a coding density of 73.8%, maintaining 49.6% of languages and 93.6% of features. The densified statistical matrix had a coding density of 70.6%, maintaining 46.2% of languages and 92.3% of features.

#### Densifying the TLI datasets

The logical and statistical TLI datasets are both larger and sparser than the GBI datasets and were thus handled differently (see Fig. 9b). They are coded for 4,259 languages at only 12.4% coding density and for 4,257 languages at only 12.1% coding density, respectively. All features are coded for at least 100 languages, but some languages are coded for very few features. Therefore, densification of the full TLI datasets focused on both the exclusion of languages to reduce the size of the dataset and on substantially increasing the proportion of coded cells. Taxonomic balance of the language sample was also desirable. For densify(), we therefore set the density mean weights to the same value, specifically density_mean_weights = list(taxonomy = 0.999, coding = 0.999). We then used prune() to select two densified sub-matrices of the resulting sub-matrices per curation (logical and statistical). One matrix preserves more languages (at the expense of only densifying the input by a factor of 1.8) while the other densifies the input more strongly, by a factor of 2.6, at the expense of including fewer languages. The larger matrices were obtained by setting scoring_function = n_data_points * coding_density * taxonomic_index and exhibit coding densities of 22.0% in the logical curation (retaining 92.0% of families, 39.4% of languages and 98.6% of features) and 22.0% in the statistical curation (retaining 91.8% of families, 39.8% of languages and 98.5% of features). Selecting the smaller matrices required a different scoring function, increasing the weight of the data retention trade-off relative to taxonomic balance and explicitly penalizing scarcely coded languages: scoring_function = n_data_points^3 * coding_density^3 * row_coding_density_min * taxonomic_index. The resulting matrices have coding densities of 33.6% in the logical curation (retaining 79.0% of families, 13.0% of languages and 96.0% of features) and 31.0% in the statistical curation (retaining 79.8% of families, 15.1% of languages and 96.4% of features).

For specific applications, it may be useful to subset the TLI datasets according to linguistic domain: grammar (here: grammatical categories and grammar), lexicon (here: lexical semantics and lexical classes) or phonology. We therefore performed additional densifications on these subsets of the TLI datasets (see Fig. 9c).

The grammar subsets were smaller but structurally similar to the full datasets, with many scarcely coded languages and low coding densities (11.1–11.7%). We thus generated one large and one small sub-matrix each for the logical and statistical curations using the same settings as for the full TLI datasets. For the large densified sub-matrices, coding densities increased to 26.1% (logical curation; retaining 87.8% of families, 30.6% of languages and 98.3% of features) and 24.2% (statistical curation; retaining 88.7% of families, 32.5% of languages and 98.7% of features). For the small densified sub-matrices, coding densities reached 44.8% (logical curation; retaining 53.4% of families, 8.2% of languages and 92.8% of features) and 40.2% (statistical curation; retaining 59.3% of families, 9.7% of languages and 93.3% of features).

The lexicon-related subsets exhibit similar numbers of languages as the grammar subsets but higher coding densities (20.3–20.6%). Hence, we only performed one densification for each curation, aiming for denser but still informative sub-matrices by setting both density mean weights to 0.999 for densify() and by defining scoring_function = n_data_points * coding_density * taxonomic_index for prune(). This yielded sub-matrices with coding densities of 39.1% (logical curation; retaining 98.0% of families, 40.0% of languages and 96.7% of features) and 39.8% (statistical curation; retaining 92.3% of families, 38.9% of languages and 96.4% of features).

Finally, in the phonology subsets, coding densities were higher (55.4–56.7%). The main aim of densification then was to prune away languages coded for only few features in a taxonomy-sensitive way. We did this by again setting both density mean weights to 0.99 for densify(). We set the scoring function of prune() to scoring_function = n_data_points * coding_density * row_coding_density_min * taxonomic_index. This yielded datasets with coding densities of 74.6% (logical curation; retaining 91.4% of families, 66.3% of languages and 86.7% of features) and 75.3% (statistical curation; retaining 83.3% of families, 64.6% of languages and 84.7% of features).

## Data Records

All inputs (external data and files documenting all curation steps), R-scripts and outputs (curated, complete and densified datasets, random diversity samples) are available on GitHub and Zenodo^36^. The repository has the following structure:

- raw-data/ external data^23–25,28,29^ (see section Methods above for details).
- curated-data/ curated datasets for GBI and TLI and the random diversity samples used for statistical tests (see following sections for a detailed description).
- plots/ figures and the R scripts used to generate them.
- scripts/ R scripts and the curation metadata used to produce the curated datasets.
  - GBI/ scripts and files used to build the curated GBI datasets and densifications.
  - TLI/ scripts and files used to build the curated TLI datasets and densifications.
  - build-curated-datasets.R invokes the scripts that build the curated datasets.
  - lang-metadata.csv manually curated taxonomic and geographic language metadata.
  - functions.R common R functions and definitions.

All the curated datasets can collectively be built by invoking the script scripts/build-curated-datasets.R. This script will invoke the relevant scripts from the respective subdirectories.

### GBI datasets

The curated data for the GBI datasets is stored in curated-data/GBI/. Within this directory the two sub-directories logicalGBI/ and statisticalGBI/ contain the logically and the statistically curated versions of GBI, respectively. The datasets are provided in a variety of formats: as a CLDF dataset, as a comma-separated *.csv table, and as a densified data matrix (files *_densified.csv in their respective sub-directories). The 500 random diversity samples are provided in 500_diversity_samples.csv.

The CLDF outputs of the logically and statistically curated GBI datasets are stored as cldf/ within the respective dataset sub-directories. They have the following structure:

- cldf/
  - languages.csv language metadata, including taxonomic and geographic information and assignment to one of the six Glottolog macroareas^46^ as well as 10 continents and 24 areas based on AUTOTYP^25^.
  - parameters.csv defines the features present in the dataset, including their classification into groups. This file additionally contains all the information necessary to reproduce the curations.
  - codes.csv defines all states in each feature.
  - values.csv stores the actual data points (features states per language).
  - modifications.csv provides motivation and description for each dataset modification.
  - sources.bib bibliography.
  - StructureDataset-metadata.json CLDF metadata file, describing each *.csv file

The scripts and curation data used to produce the curated versions are in scripts/GBI/. The CLDF and main tabular output for GBI is produced by the script build-datasets.R. It reads the raw Grambank data (contained in raw-data at the root of the repository) and writes the curated data output to curated-data/GBI/. The curation code is controlled by the two curation files decisions-log.csv and feature-recode-patterns.csv (Fig. 2a). The file decisions-log.csv documents all modifications, and the file feature-recode-patterns.csv records and defines every feature associated with either the original Grambank dataset or one of the GBI datasets. The curation data is also stored in the CLDF output. The decisions-log.csv file corresponds to the CLDF file modifications.csv. Relevant rows of the file feature-recode-patterns.csv correspond to the parameters.csv file of the CLDF datasets. The R-script test-dependencies.R reproduces the 500 random diversity samples (500_diversity_samples.csv within curated-data/GBI/). These are the samples that were used to assess the dependencies for the statistical curation. The script also reproduces and validates the relevant computations, i.e. the entries in decisions-log.csv that document the dependency test statistics. Finally, the R script densify-datasets.R performs matrix densification of the full logical and statistical GBI datasets, producing the densified datasets and storing them in the respective dataset sub-directory under curated-data/GBI.

### TLI datasets

The curated data for the TLI datasets is stored in curated-data/TLI/. This sub-directory, as well as the accompanying scripts in scripts/TLI/ are structured similarly to the above, with some important differences.

The scripts directory scripts/TLI/ contains an additional script compile-external-input.R which is invoked as the first step of the build process. This script performs the data fusion of WALS, AUTOTYP, Lexibank, and PHOIBLE data. The feature definitions for PHOIBLE are provided in the file phoible-feature-definitions.csv alongside the script. The fused data is stored in the file compiled_external_input_features.csv in the curated data directory curated-data/TLI/. The script build-datasets.R subsequently uses this fused data to produce the curated datasets. This script is controlled by the curation files using the curation files decisions-log.csv and feature-recode-patterns.csv in the script directory (Fig. 2b). The curated data output is stored as a CLDF dataset for logical and statistical TLI within the respective sub-folders of curated-data/TLI/. The full language-feature matrices are stored as comma-separated files in the full/ sub-directory of the respective output location. The script test-dependencies.R reproducibly generates the 1000 random diversity samples and stores them within the curated-data/TLI/ directory as 1000_diversity_samples.csv. These are the samples that were used to assess the expectations for the statistical curation. The script also reproduces and validates the relevant computations, i.e. the entries in decisions-log.csv that document the dependency test statistics. Lastly, the script densify-datasets.R performs matrix densification on the full TLI-logical and TLI-statistical datasets, as well as their domain-specific subsets. We performed further densifications for data on phonological and lexical features. In addition, we performed two separate densifications — producing a large and a small matrix — on the full dataset and the grammar-only dataset. The densified data is appropriately named and stored within the relevant sub-directory of curated-data/TLI/logicalTLI and curated-data/TLI/statisticalTLI, respectively.

## Technical Validation

To assess the validity and illustrate use-cases of our data curations, we performed two experiments.

1. We assessed the extent to which the logical and statistical curations resolve biases in diversity estimates that average across all available features.
2. We assessed overall signal preservation after densification of curated datasets.

### Experiment 1: Curations avoid biases induced by dependencies between features

Statistical applications assuming that all input features track independent characteristics of languages will provide biased results when this assumption is violated. Here, we use an analysis of local typological diversity that requires independent features to show the presence of such biases.

**Fig. 10:**
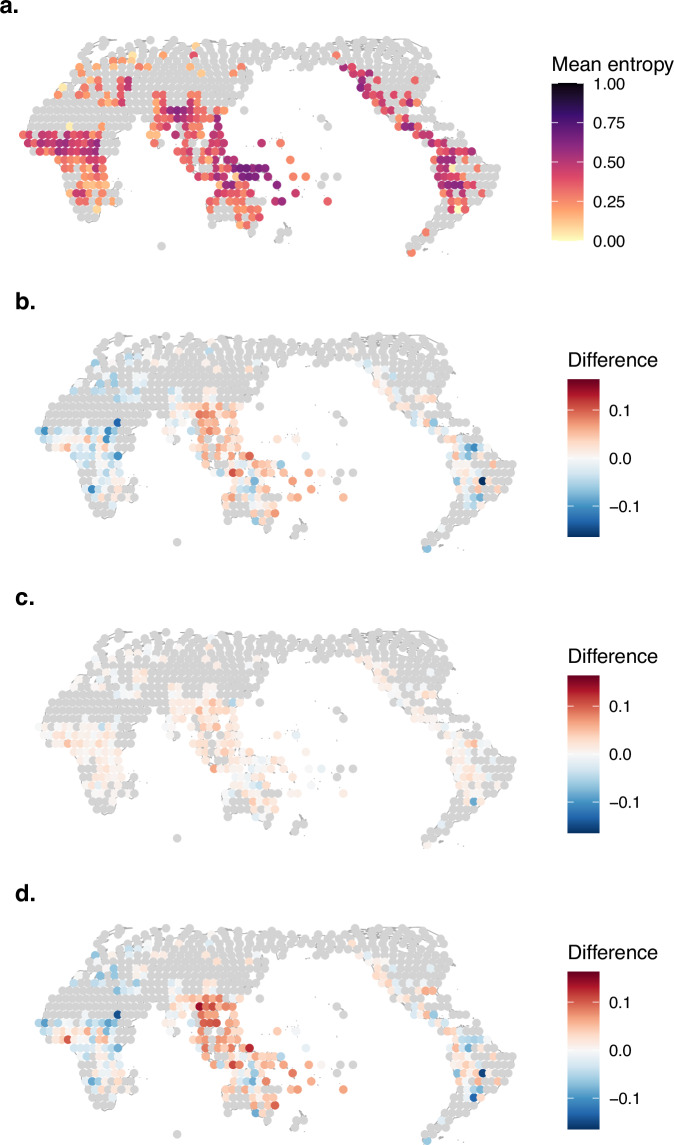
(**a**) Global distribution of local typological diversity (mean feature entropy) in GBI-statistical. (**b-d**) Differences in mean entropy across the Grambank/GBI curations, with shades of red indicating entropy increases (i.e., removal of dependencies resulted in higher regional typological diversity) and shades of blue indicating entropy decreases. (**b**) GBI-logical vs. Grambank (external input). (**c**) GBI-statistical vs. GBI-logical. (**d**) GBI-statistical vs. Grambank (external input).

We created a regular spherical grid on continents and islands^51^ and then associated each language to its nearest grid point for GBI-statistical. For each grid point with at least two languages, we computed the normalized entropy of the distribution of non-{NA} and non-{?} feature states of the languages associated with this grid point for each feature, using the entropy() function of the posterior package in R^52^. To reduce biases resulting from data sparsity, we only did this when more than 50% of languages were coded for the relevant feature at the grid point. Figure 10a shows the arithmetic mean of all normalized entropies at each grid point. Grid points with high average entropies thereby indicate high local diversity in terms of linguistic structures. Low average entropies represent more homogeneity in terms of locally attested linguistic structures.

For these measures to be meaningful, the features must be independent. Otherwise, the method will underestimate diversity at grid points where many languages are invalidly treated as showing the same states across several features. For instance, if at a certain grid point several languages lack gender, entropies in gender-related features will differ depending on whether these languages are encoded as lacking each of the up to 13 specific manifestations of gender (original Grambank setup, see Table 2) or whether a meta-feature is introduced that tracks lack of gender in a single feature without repeating this signal across many features (GBI-logical): *ceteris paribus*, the original setup will yield lower entropy values than curated GBI-logical.

Figure 10b-d quantifies the difference in mean entropies in each grid point across the original Grambank data, GBI-logical and GBI-statistical. This visualizes the impact of the logical and statistical curation steps. We find that the original Grambank dataset strongly underestimates morphosyntactic diversity in South-East Asia. This is most likely related to the way that degree of fusion is handled. In the original Grambank dataset, there are many features that distinguish between morphological marking (*on the verb*, *on the noun* etc.) as opposed to marking by phonologically free elements. If these are used in their original state, many languages of Southeast Asia are likely to appear very similar since they tend to lack morphological marking completely. When these features track the same grammatical category (e.g. tense), they have been reduced in GBI datasets, mitigating this effect.

This confirms that uncurated datasets can generate patterns that are spuriously induced by undetected dependencies.

### Experiment 2: Densified data subsets preserve typological signal

Densified datasets should expand the usability of data of very large and/or sparsely coded datasets to applications that can only handle inputs of smaller scale and/or with higher data coverage. However, sub-sampling input data inevitably leads to data loss. For a densified sub-matrix to be useful, it should preserve signals encoded in the full matrix to the greatest extent possible.

**Fig. 11:**
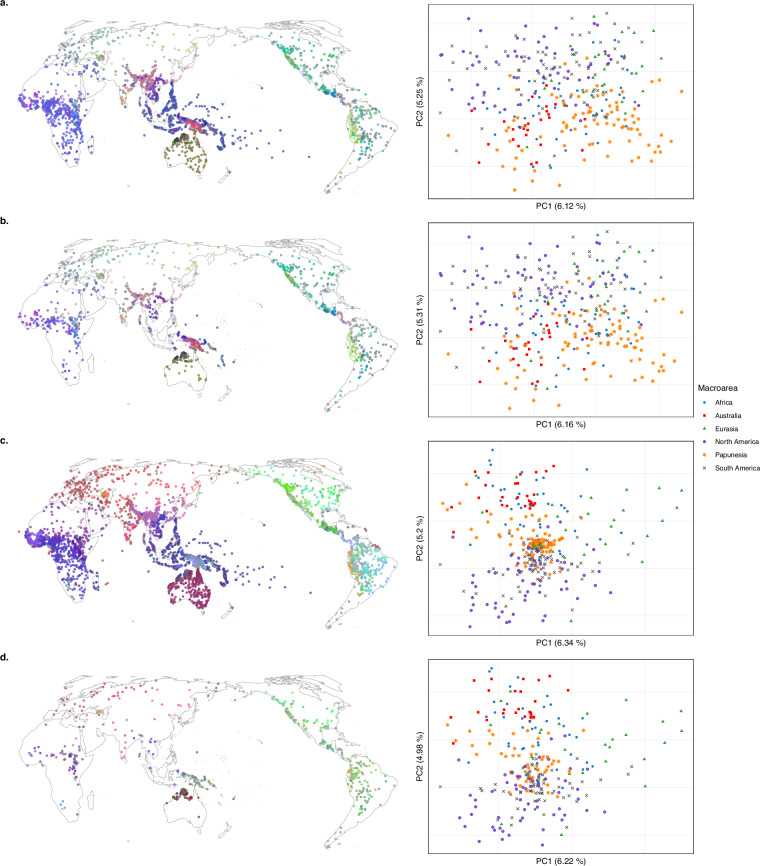
Results of Principal Component Analysis on family proportion data. Units of analysis are families and isolates. Left: Family-wise projection of PC1-PC3 into RGB color space. Languages are colored by the value of their family. Similar shades of color within a map indicate typological similarity between families. Identical color indicates identity of family. Right: Families by macroarea in PC1-PC2 space, colored by region (not PCs). (**a**) Full GBI-logical dataset (R2 of PC1–PC3 = 15.14%). (**b**) Densified GBI-logical dataset (R2 of PC1–PC3 = 15.14%). (**c**) Full GBI-logical dataset (R2 of PC1–PC3 = 15.40%). (**d**) Small densified GBI-logical dataset (R2 of PC1–PC3 = 15.07%).

To test this, we compared global typological similarity across phylogenetic units between the logically curated GBI and TLI datasets and their densified subsets. For this, we performed Principal Component Analysis (PCA) with NIPALS imputation (non-linear estimation through iterative partial least squares)^53^ using the pca() function of the pcaMethods^54^ package in R. Principal Component Analysis requires continuous data but not statistical independence of features. We therefore used the logical curations of our datasets in this experiment. To control for phylogenetic non-independence, we aggregated the data at the level of language families, computing the proportion of languages in each family with each feature state. Since one proportion per feature is always redundant, we omitted calculating proportions for the most frequently attested feature state of each feature. The resulting table of per-family proportions served as the input for the PCA. Adopting a visualization approach pioneered in dialectology^55^ we visualized the principal components by assigning each family a value in RGB-color space (i.e., a color) mapped to the first three PCs, concretely: PC1 to shades of red, PC2 to shades of green, and PC3 to shades of blue. These shades of color were then mapped to the languages in the sample representing each family (left panels of Fig. 11). Similar shades of color within a map thus express typological similarity while identical colors show that languages below the same language family. To facilitate the comparison of patterns across maps, we additionally plotted each family by its geographical macroarea into a space defined by PC1 and PC2 (right panels of Fig. 11). The PCA-outputs retrieved from the full and densified GBI dataset and from the full and small densified TLI dataset show that the language-feature subsets of the full matrices generated by our densifications yield strikingly similar results (correlations for GBI full vs. densified are at r = 99.9% for PC1, PC2 and PC3; correlations for TLI full vs. small densified they are at r = 98.2% for PC1, at r = 98.3% for PC2 and at r = 53.2% for PC3). They also show that the aggregated global family-level patterns encoded within the GBI and full TLI datasets are similar for certain macro-patterns albeit not identical.

Results suggest that densification preserves signal to a sufficient extent. This validates the use of our curations when a reduction of NA proportions or sample size is needed for a specific application.

## Usage Notes

The full datasets presented here can be accessed by a wide range of data processing programs. Specific libraries facilitating use of data in CLDF format are available in R (rcldf)^56^ and Python (pycldf)^57^. The presented densified subsets of the data are available as .csv-files in language-feature matrix format. The R package densify^35^ allows to generate data subsets with other attributes, should the densifications provided here prove unsuitable for particular applications.

The statistically curated datasets can be modified to comply with different levels of stringency. This requires adjusting thresholds in the test-dependencies.R scripts, consequently modifying the relevant helper columns in the decisions-log.csv files. Changes to curation decisions must then be manually made and implemented in the file feature-recode-patterns.csv.

The workflow for data curation can also be applied to other datasets. This requires careful assessment of the data to create appropriate decisions-log.csv and feature-recode-patterns.csv files. Data and scripts made available here can serve as templates to facilitate this process.

## Data Availability

All curated datasets as well as all files and scripts used to produce them and all figures presented here are hosted in a GitHub repository (https://github.com/annagraff/crossling-curated), available under a CC-BY 4.0 licence, and archived on Zenodo (10.5281/zenodo.14606326)^36^. All data processing is implemented in R. The current repository release is v.1.0.

## References

[1] Wilkinson, M. D. *et al*. The FAIR Guiding Principles for scientific data management and stewardship. *Sci. Data* **3**, 160018 (2016). 26978244 10.1038/sdata.2016.18 PMC4792175

[2] International Organization for Standardization. Code for the representation of languages. Part 3: Alpha-3 code for comprehensive coverage of languages (2007).

[3] Forkel, R. & Hammarström, H. Glottocodes: Identifiers linking families, languages and dialects to comprehensive reference information. *Semantic Web* **13**, 917–924 (2022).

[4] Forkel, R. *et al*. Cross-Linguistic Data Formats, advancing data sharing and re-use in comparative linguistics. *Sci. Data* **5**, 180205 (2018). 30325347 10.1038/sdata.2018.205 PMC6190742

[5] European Organization for Nuclear Research & OpenAIRE. Zenodo. 10.25495/7GXK-RD71 (2013).

[6] Winter, B. & Grice, M. Independence and generalizability in linguistics. *Linguistics* **59**, 1251–1277 (2021).

[7] Roberts, S. & Winters, J. Linguistic Diversity and Traffic Accidents: Lessons from Statistical Studies of Cultural Traits. *PLOS ONE* **8**, e70902 (2013). 23967132 10.1371/journal.pone.0070902 PMC3743834

[8] Bromham, L., Hua, X., Cardillo, M., Schneemann, H. & Greenhill, S. J. Parasites and politics: why cross-cultural studies must control for relatedness, proximity and covariation. *R. Soc. Open Sci.* **5**, 181100 (2018). 30225088 10.1098/rsos.181100 PMC6124128

[9] Dryer, M. S. Large Linguistic Areas and Language Sampling. *Stud. Lang. Int. J. Spons. Found. “Foundations Lang.* **13**, 257–292 (1989).

[10] Gavin, M. C. *et al*. Toward a Mechanistic Understanding of Linguistic Diversity. *BioScience* **63**, 524–535 (2013).

[11] Miestamo, M., Bakker, D. & Arppe, A. Sampling for variety. *Linguist. Typology* **20**, 233–296 (2016).

[12] Dunn, M., Greenhill, S. J., Levinson, S. C. & Gray, R. D. Evolved structure of language shows lineage-specific trends in word-order universals. *Nature* **473**, 79–82 (2011). 21490599 10.1038/nature09923

[13] Matsumae, H. *et al*. Exploring correlations in genetic and cultural variation across language families in northeast Asia. *Sci. Adv.* **7**, eabd9223 (2021). 34407936 10.1126/sciadv.abd9223 PMC10964961

[14] Ranacher, P. *et al*. Contact-tracing in cultural evolution: a Bayesian mixture model to detect geographic areas of language contact. *J. R. Soc. Interface* **18**, 20201031 (2021). 34376092 10.1098/rsif.2020.1031 PMC8355670

[15] Skirgård, H. *et al*. Grambank reveals the importance of genealogical constraints on linguistic diversity and highlights the impact of language loss. *Sci. Adv.* **9**, eadg6175 (2023). 37075104 10.1126/sciadv.adg6175 PMC10115409

[16] Bickel, B. & Nichols, J. Oceania, the Pacific Rim, and the Theory of Linguistic Areas. *Annu. Meet. Berkeley Linguist. Soc*. 3–15 10.3765/bls.v32i2.3488 (2006).

[17] Hammarström, H. & O’Connor, L. Dependency-sensitive typological distance. in *Approaches to Measuring Linguistic Differences* (eds. Borin, L. & Saxena, A.) 329–352 10.1515/9783110305258.329 (De Gruyter Mouton, 2013).

[18] Bickel, B. Large and ancient linguistic areas. in *Language Dispersal, Diversification, and Contact* (eds. Crevels, M. & Muysken, P.) 0 10.1093/oso/9780198723813.003.0005 (Oxford University Press, 2020).

[19] Greenberg, J. H. Some Universals of Grammar with Particular Reference to the Order of Meaningful Elements. in *Universals of Language* 58–90 10.1515/9781503623217-005 (The M.I.T. Press, Cambridge, Massachusetts, 1963).

[20] Dryer, M. S. Why statistical universals are better than absolute universals. *Pap. 33rd Reg. Meet. Chic. Linguist. Soc*. 1–23 (1998).

[21] Bickel, B. Areas and Universals. in *The Cambridge Handbook of Areal Linguistics* (ed. Hickey, R.) 40–54 10.1017/9781107279872.004 (Cambridge University Press, 2017).

[22] Round, E. R. Big data typology and linguistic phylogenetics: design principles for valid datasets. in (Manchester, 2013).

[23] Skirgård, H. *et al*. Grambank v1.0. *Zenodo*10.5281/zenodo.7844558 (2023).

[24] Dryer, M. & Haspelmath, M. WALS Online (v2020.3). *Zenodo*10.5281/zenodo.7385533 (2013).

[25] Bickel, B. *et al*. The AUTOTYP database. *Zenodo*10.5281/zenodo.7976754 (2023).

[26] Witzlack-Makarevich, A., Nichols, J., Hildebrandt, K. A., Zakharko, T. & Bickel, B. Managing AUTOTYP Data: Design Principles and Implementation. in *The Open Handbook of Linguistic Data Management* (eds. Berez-Kroeker, A. L., McDonnell, B., Koller, E. & Collister, L. B.) 631–642 10.7551/mitpress/12200.003.0061 (The MIT Press, 2022).

[27] List, J.-M. *et al*. Lexibank, a public repository of standardized wordlists with computed phonological and lexical features. *Sci. Data* **9**, 316 (2022).

[28] List, J.-M. *et al*. Lexibank Analysed. *Zenodo*10.5281/zenodo.7836668 (2023).

[29] Moran, S. & McCloy, D. PHOIBLE https://github.com/phoible/dev/commit/7030ae02863f0e1ddaf67f0f950c0ea1477cd4ee (2023).

[30] Baum, D. A. & Smith, S. D. *Tree Thinking: An Introduction to Phylogenetic Biology*. (Roberts, Greenwood Village, Colo, 2012).

[31] Cathcart, C. & Bickel, B. Linguistic evolution in time and space: addressing the methodological challenges (2024).

[32] Freudenstein, J. V. Characters, States and Homology. *Syst. Biol.* **54**, 965–973 (2005). 16385777 10.1080/10635150500354654

[33] Bickel, B. Absolute and statistical universals. in *The Cambridge Encyclopedia of the Language Sciences* (ed. Hogan, P. C.) 77–79 (Cambridge University Press, Cambridge, 2011).

[34] Hammarström, H., Forkel, R., Haspelmath, M. & Bank, S. glottolog/glottolog: Glottolog database 5.0. *Zenodo*10.5281/zenodo.10804357 (2024).

[35] Graff, A., Lischka, M., Zakharko, T., Furrer, R. & Bickel, B. densify: An R package to reduce empty cells in data frames of typological linguistic data. *J. Open Source Softw* **9**, 7024 (2024).

[36] Graff, A. *et al*. GBI and TLI: Release version, *Zenodo*, 10.5281/zenodo.14606326 (2025).

[37] Corbett, G. G. Number of Genders (v2020.3). *The World Atlas of Language Structures Online*10.5281/zenodo.7385533 (2013).

[38] Corbett, G. G. Sex-based and Non-sex-based Gender Systems (v2020.3). *The World Atlas of Language Structures Online*10.5281/zenodo.7385533 (2013).

[39] Anderson, G. D. S. The Velar Nasal (v2020.3). *The World Atlas of Language Structures Online*10.5281/zenodo.7385533 (2013).

[40] R Core Team. R: A language and environment for statistical computing. R Foundation for Statistical Computing (2024).

[41] Bickel, B. & Zakharko, T. Recodings of WALS Online data. *GitHub* https://github.com/IVS-UZH/WALS-recodings (2018).

[42] Plank, F. & Filimonova, E. Rara and the Universals Archive. https://typo.uni-konstanz.de/rara/ (2000).

[43] Jäger, G. & Wahle, J. Phylogenetic Typology. *Front. Psychol*. **12** (2021). 10.3389/fpsyg.2021.682132 PMC8326798 34349702

[44] Bickel, B. Statistical modeling of language universals. **15**, 401–413 (2011).

[45] Murayama, K., Pekrun, R. & Fiedler, K. Research Practices That Can Prevent an Inflation of False-Positive Rates. *Personal. Soc. Psychol. Rev.* **18**, 107–118 (2014). 10.1177/1088868313496330 23965303

[46] Hammarström, H. & Donohue, M. Some Principles on the Use of Macro-Areas in Typological Comparison. *Lang. Dyn. Change* **4**, 167–187 (2014).

[47] Cysouw, M. Against implicational universals. **7**, 89–101 (2003).

[48] Cohen, J. *Statistical Power Analysis for the Behavioral Sciences*10.4324/9780203771587 (Routledge, New York, 1988).

[49] Navarro, D. lsr: Companion to ‘Learning Statistics with R’. (2021).

[50] Sullivan, G. M. & Feinn, R. Using Effect Size—or Why the P Value Is Not Enough. *J. Grad. Med. Educ.* **4**, 279–282 (2012). 23997866 10.4300/JGME-D-12-00156.1 PMC3444174

[51] Derungs, C., Köhl, M., Weibel, R. & Bickel, B. Environmental factors drive language density more in food-producing than in hunter–gatherer populations. *Proc. R. Soc. B Biol. Sci.* **285**, 20172851 (2018). 10.1098/rspb.2017.2851 PMC6125899 30135170

[52] Bürkner, P.-C. *et al*. posterior: Tools for Working with Posterior Distributions. (2024).

[53] Wold, H. Estimation of principal components and related models by iterative least squares. *Multivar. Anal.* **391**, 420 (1966).

[54] Stacklies, W., Redestig, H., Scholz, M., Walther, D. & Selbig, J. pcaMethods—a bioconductor package providing PCA methods for incomplete data. *Bioinformatics* **23**, 1164–1167 (2007). 17344241 10.1093/bioinformatics/btm069

[55] Nerbonne, J., Heeringa, W. & Kleiweg, P. Edit distance and dialect proximity. in *Time Warps, String Edits and Macromolecules: The theory and practice of sequence comparison* (eds. Sankoff, D. & Kruskal, J.) vol. 15 (CSLI Press Stanford, 1999).

[56] Greenhill, S. J. rcldf - The R library for reading CLDF files (2024).

[57] Forkel, R., Bank, S., Greenhill, S. J., Rzymski, C. & Kaiping, G. pycldf. *Zenodo*10.5281/zenodo.3355430 (2019).

