# Author Correction: Curating global datasets of structural linguistic features for independence

**DOI:** 10.1038/s41597-025-04592-x

**Published:** 2025-02-12

**Authors:** Anna Graff, Natalia Chousou-Polydouri, David Inman, Hedvig Skirgård, Marc Lischka, Taras Zakharko, Chiara Barbieri, Balthasar Bickel

**Affiliations:** 1 Institute for the Interdisciplinary Study of Language Evolution (ISLE), University of Zurich, Zürich, Switzerland; 2 Department of Evolutionary Biology and Environmental Studies, University of Zurich, Zürich, Switzerland; 3 Institute for Mediterranean Studies, Foundation for Research and Technology - Hellas, Rethymno, Greece; 4 Department of Linguistic and Cultural Evolution, Max Planck Institute for Evolutionary Anthropology, Leipzig, Germany; 5 Institute of Mathematics, University of Zurich, Zürich, Switzerland; 6 Dipartimento di Scienze della vita e dell’ambiente, Università degli Studi di Cagliari, Cagliari, Italy

**Keywords:** Anthropology, Anthropology, Evolution of language, Anthropology, Anthropology, Evolution of language

Correction to: *Scientific Data* 10.1038/s41597-024-04319-4, published online 18 January 2025

In this article reference 29 incorrectly read, ‘PHOIBLE 2.0. (Max Planck Institute for the Science of Human History, Jena, 2019)’ and should have read, ‘Moran, S. & McCloy, D. PHOIBLE https://github.com/phoible/dev/commit/7030ae02863f0e1ddaf67f0f950c0ea1477cd4ee (2023)’. The original article has been corrected.

